# STARR with CONTOUR® TRANSTAR™ device for obstructed defecation syndrome: one-year real-world outcomes of the European TRANSTAR registry

**DOI:** 10.1007/s00384-014-1836-8

**Published:** 2014-02-21

**Authors:** G. Ribaric, A. D’Hoore, G. Schiffhorst, E. Hempel, The TRANSTAR Registry Study Group

**Affiliations:** 1 Clinical and Medical Affairs, Ethicon Endo-Surgery (Europe) GmbH, European Surgical Institute, Norderstedt/Hamburg, Germany; 2 Department of Abdominal Surgery, University Hospital Leuven, Leuven, Belgium; 3 Department of Statistics and Biometry, IGES Institute GmbH, Berlin, Germany; 4 Clinical Data Management, IGES Institute GmbH, Berlin, Germany; 5 Clinical and Medical Affairs, Ethicon Endo-Surgery (Europe) GmbH, MD&D EMEA (Europe, Middle East, Africa), Johnson & Johnson, Hummelsbütteler Steindamm 71, 22851 Norderstedt, Germany

**Keywords:** STARR, Obstructive defecation syndrome, Constipation, Internal rectal prolapse, Rectocele, TRANSTAR

## Abstract

**Purpose:**

Stapled transanal rectal resection (STARR) in patients with obstructive defecation syndrome (ODS) is limited by the capacity of the circular stapler used. This prospective cohort study was conducted to assess real-world clinical outcomes of STARR with the new CONTOUR® TRANSTAR™ device, shortly named TRANSTAR, at 12 months postoperatively.

**Methods:**

From January 2009 to January 2011, consecutive patients who underwent TRANSTAR in 22 European colorectal centers were enrolled in the study. Functional outcomes and quality of life were assessed by the changes in a number of scoring systems (Knowles-Eccersley-Scott-Symptom (KESS) score, ODS score, St. Mark’s score, Euro Quality of Life-5 Dimension (EQ-5D) score, and Patient Assessment of Constipation—Quality of Life (PAC-QoL) score), at 12 months as compared to baseline. All complications were recorded and analyzed.

**Results:**

A total of 100 patients (98 % female), mean age 60 years, were entered in the study. Statistically significant improvements were seen in the KESS (median 18 vs. 6; *p* < 0.01), ODS (median 15 vs. 4; *p* < 0.01), and PAC-Qol scores (median 2.10 vs. 0.86; *p* < 0.01). St. Mark’s and EQ-5D scores improved nonsignificantly. Complications were reported in 11 % of patients, including bleeding (5 %), staple line complications (3 %), urinary retention (2 %), and persistent pain (1 %). No major complications or mortality occurred.

**Conclusion:**

TRANSTAR facilitated a tailored, real circumferential full-thickness rectal resection, leading to improved patient functional and quality of life outcomes at 12 months postoperatively. It represents a safe and effective treatment for ODS in local clinical practice, although the sustainability of real-world results needs to be proven in the long-term follow-up.

## Background

Obstructive defecation syndrome (ODS) is a poorly understood condition, characterized by the urge to defecate but an impaired ability to evacuate the rectum [1, 2]. The symptoms include frequent visits to the toilet with unsuccessful evacuation attempts, prolonged straining, anorectal discomfort or pain, fresh rectal bleeding, a sensation of incomplete evacuation, and the need for manual assistance [2, 3]. Structural abnormalities such as rectocele, enterocele or genital prolapse, and non-relaxing puborectalis may be associated with ODS, or coexist [4]. However, the internal rectal mucosal prolapse and rectal intussusception have been recognized as major pathomorphological determinants of ODS, and consequently, a plethora of abdominal, vaginal, and laparoscopic surgical procedures have been used to correct the underlying condition [5–8].

As a minimally invasive technique for achieving a full-thickness resection of the distal rectum, stapled transanal rectal resection (STARR) has been proposed for the treatment of ODS by performing two firings of the PPH-01 circular stapler (Ethicon Endo-Surgery, Inc., Cincinnati, OH), which was originally designed for use in stapled hemorrhoidopexy [9, 10]. In 2006, the National Institute for Health and Care Excellence (NICE) in the United Kingdom issued clinical guidance on STARR concluding that the current evidence for its safety and efficacy for ODS did not appear adequate for this procedure to be used without special arrangements for consent and for audit or research [4].

In response to the NICE guidance, the European STARR registry was established through the collaboration between the manufacturer, Ethicon Endo-Surgery (Europe) GmbH, Norderstedt, Germany, and the colorectal societies in France, Germany, Italy, and the UK. The real-world outcomes from this registry have recently been published, reinforcing the conclusions from single- and multicenter trials that STARR provides a significant patient benefit, at least in the short term, and can be performed safely and without major morbidity [11–15]. Based on the published evidence, NICE issued its updated clinical guidance on STARR in 2010, concluding that: “The current evidence on the safety and efficacy of stapled transanal rectal resection (STARR) for obstructed defecation syndrome (ODS) is adequate for this condition which can significantly affect the quality of life. The procedure may therefore be used with normal arrangements for clinical governance, consent and audit” [5].

Despite these conclusions on its proven safety and efficacy by the leading independent scientific institute (NICE), the limitations of STARR, mostly related to the design of the circular stapler used, were recognized by a broad surgical community. During this transanal procedure, the rectal wall resection is performed “blind” after the insertion of the circular stapler in the lower rectum, which might cause the entrapment of the device in the dilator and increase the risk of rectal perforation [2, 16]. Furthermore, the volume of resection was dependent on the capacity of the circular stapler housing rather than on the size of the underlying rectal intussusceptions, without the possibility for surgeons to tailor the extent of the rectal resection to the size of the prolapse [2, 17].

As a response to the observed difficulties, a new stapling device, the CONTOUR® TRANSTAR™ curved cutter-stapler (Ethicon Endo-Surgery, Inc., Cincinnati, OH), was designed to improve STARR by allowing the tailored circumferential correction of the internal rectal prolapse under continuous visual control. Following the stapler development, the innovative transanal stapling technique STARR with CONTOUR® TRANSTAR™, shortly named TRANSTAR, has been proposed for the treatment of ODS [2, 16, 17] and was selectively launched in European countries where the health care decision-makers explicitly recommended the broad assessment of transanal stapling procedures in local clinical practice [4, 5, 7].

A major challenge in assessing local practice reported in the literature previously, concluded that the variation phenomenon is so widespread and robust in local practice that it can be found almost anywhere where health care researchers look for it: between the care of the very young and the very old, between inpatient and outpatient settings, and between several geographic regions [18]. The aim of this study was to assess the short-term safety and effectiveness of TRANSTAR in the real-world clinical environment where the variation phenomenon exists.

## Methods

### Study design and setting

This registry was designed as a prospective, real-world, multicenter cohort study to capture short-term safety, functional, and patient-reported outcomes with the use of TRANSTAR, taking into account the contemporary variations in local surgical practice throughout Europe. The anonymous patient data were entered in the local investigating centers according to the study protocol and into standardized data collection forms (clinical record forms). These were subsequently adapted and transcribed into electronic format for web-based data collection to allow patient data to be collected at the same time in all centers. A web-based interface was used to facilitate simultaneous data entry, concurrently merging the data into a single European TRANSTAR Registry data set. The online interface and data entry were monitored and managed by an independent clinical trial and statistical support agency (CSG-Clinische Studien Gesellschaft mbH, Berlin, Germany, and IGES Institut GmbH, Berlin, Germany). A European steering group was established to oversee the data collection, analysis, and interpretation. The study was conducted in accordance with applicable local and national laws and regulations, with the Medical Devices Directive 93/42/EEC (MDD) Annex X, the European Standard EN ISO 14155-1, and with the Declaration of Helsinki and all its amendments. The central ethical committee of the Board of Physicians, Berlin, Germany, approved the study, and each individual investigating center obtained approval from its local ethical committee. The patient data collection was the responsibility of the investigating surgeons, who certified that all patients gave written informed consent to take part in the study. This study was registered online in the publicly available trials database clinicaltrials.govas “International Multicenter Prospective Transtar Registry” with the identification number NCT00909116. The STROBE Statement-checklist was used to report the results of this cohort study.

In total, 22 colorectal surgeons from Austria, Belgium, France, Germany, Italy, Portugal, Spain, and Switzerland enrolled patients in the electronic database. All investigating surgeons were experienced in the original STARR technique and required to complete a specific training in TRANSTAR at the manufacturer surgical education center in Norderstedt, Germany. Following the training, the investigators’ first TRANSTAR cases performed in the local setting were monitored by the members of STARR Pioneers, a surgical expert group created by Ethicon Endo-Surgery (Europe) GmbH, Norderstedt, Germany, to support continuous education and proctorship of the transanal stapling techniques for anorectal prolapse. In this way, the investigating surgeons gained proficiency with the new stapling device before starting to enroll the patients in this study. The clinical outcomes of interest were preoperative patient clinical status, surgical complications, postoperative functional outcomes, and quality of life results. The longitudinal follow-up assessments were scheduled 6 weeks and 6 and 12 months after surgery, with the aim to perform the analysis of the results at 12 months follow-up as compared to baseline.

### Participants

Consecutive patients with ODS treated with TRANSTAR in the 22 investigating centers across Europe were included in this cohort. Inclusion recommendations in the study protocol were aligned with the recently published patient selection algorithm for STARR [19]. Briefly, the patients were selected for the surgery on the basis of recognized clinical symptoms of ODS (e.g., frequent visits to the toilet with unsuccessful evacuation attempts, prolonged straining, anorectal discomfort or pain, a sensation of incomplete evacuation, and the need for manual assistance) associated with evidence of rectal pathology such as rectocele and/or internal rectal prolapse confirmed by clinical examination with proctoscopy and/or diagnostic imaging (defecography or MRI). An adequate anal sphincter function was assessed at least with a digital rectal examination.

Exclusion criteria included patients with a contraindication to general anesthesia, physical or psychological problems precluding data collection, and coexisting inflammatory or septic conditions of the rectum. Recommendations were made surrounding preoperative investigation, patient preparation, operative technique, and postoperative care.

However, the ultimate decision-making was left to individual investigators in line with local policy.

### Surgical technique and postoperative recommendations

Recommendations for the surgical technique were based on the recently published description of TRANSTAR [2, 16]. Briefly, the preoperative preparation included one or two phosphate enemas the morning of surgery, routine deep vein thrombosis prophylaxis, and perioperative broad spectrum antibiotics. General or regional anesthesia was used based on the individual surgeon’s preference. The patient was placed in the lithotomy position with the hips in hyperflexion. An initial examination was undertaken to confirm the presence and extent of the internal rectal prolapse and rectocele, and to confirm the absence of coexistent pathology. The CONTOUR® TRANSTAR™ stapling kit (Ethicon Endo-Surgery Inc., Cincinnati, OH, USA) was opened and the circular anal dilator (CAD) gently introduced and fixed to the perianal skin with four cardinal sutures. A swab was inserted and gently pulled outward to visualize the apex of the intussusception. Finally, the TRANSTAR technique was performed in three steps: step 1: parachute suture placement, step 2: opening of the prolapse, and step 3: circumferential resection. Regarding the postoperative period, an easily digestible diet from the first postoperative day to the end of hospitalization and administration of analgesics as required were recommended. The thrombotic prophylaxis was given until discharge from the hospital.

### Variables

Functional and patient-reported outcomes were measured by using disease-specific and generic-scoring systems. The primary study endpoint was defined as the change in the validated KESS (Knowles-Eccersley-Scott-Symptom) constipation score at 12 months postoperatively as compared to baseline [20]. Accordingly, the secondary study endpoints were defined as the change in the ODS (Longo) constipation score, St. Mark’s (Vaizey) incontinence score, and disease-specific PAC-QoL score (Patient Assessment of Constipation—Quality of Life), as well as the generic quality of life instruments EQ-5D (Euro Quality of Life-5 Dimension) and EQ-5D VAS (Visual Analog Scale), between baseline and 12 months follow-up [15, 21–26]. Monitoring of complications started in the hospital and was followed up in the outpatient setting until 12 months after surgery.

#### Functional scores

##### Knowles-Eccersley-Scott-Symptom (KESS) score

The KESS score has been statistically validated as a tool for distinguishing constipated patients with a proven pathophysiologic abnormality, from those in whom physiologic investigations were normal, predicting 96 % of cases correctly [20]. The overall KESS score is derived by summation of its individual components to give a maximum score of 40. A higher score indicates more severe symptoms. There are no known biological or physiological markers for the severity of constipation, so it is unclear what the significance of an overall KESS score is. However, in its validation report, constipated patients with a median KESS score of 20 (range, 11–35) were significantly different from healthy controls presenting with a median score of 2 (range, 0–6; *P* < 0.0001), with no overlap between the scores of constipated and control patients [20]. Moreover, using a cut-off criterion of ≥10, the overall KESS score had a 100 % sensitivity (95 % confidence interval (CI) = 95–100 %) and a 100 % specificity (95 % CI = 63–100 %) to differentiate clearly constipated patients from healthy controls [20]. Accordingly, the KESS score was used as a primary functional endpoint to monitor the clinical effectiveness of TRANSTAR in this study.

##### Obstructive Defecation Score (ODS) (A. Longo)

The Longo’s ODS score is an unvalidated tool for assessing functional symptoms of outlet obstruction which was frequently used in previously published trials on STARR. Nine symptoms of obstructive defecation are scored on a scale of 0 to 4, and accordingly, the maximum ODS score is 36. The total ODS score is derived by summation of the individual components with a higher score indicating more severe symptoms [2, 15].

##### St. Mark’s incontinence score (Vaizey C.J.)

St. Mark’s score is a validated scoring system that combines components of incontinence disorder (fecal incontinence for solid or liquid stool or for flatus alone, as well as frequency and quantity of stool lost), with an assessment of defecatory urgency and the need to take antidiarrheal medication. Impaired incontinence may be passive—that is, without the patient’s awareness, or urgent—that is, the inability to defer defecation, and both of these are reflected in the scale. An indication of the effect of incontinence on lifestyle (including the need to use pads or plugs and the ability to perform work and leisure activities) is also taken into account in the validated St. Mark’s score [21]. The total score is derived by summation of the individual components, giving a maximum score of 24, with a higher score indicating worse function [21].

#### Quality of life instruments

##### Patient Assessment of Constipation Quality of Life (PAC-QoL) score

The PAC-QoL score is a comprehensive assessment of the burden of constipation on patients’ everyday functioning and well-being [22]. It is a validated, internally consistent, reproducible questionnaire that was developed to evaluate constipation over time. The 28 items of the PAC-QoL form four subscales (worries and concerns, physical discomfort, psychosocial discomfort, and satisfaction), as well as a total score. The PAC-QoL scale scores are significantly associated with abdominal pain (*p* < 0.001) and constipation severity (*p* < 0.05). PAC-QoL score was assessed according to its original derivations, with an improvement in QoL expressed by a decrease in numerical value of the score [22]. PAC-QoL is a disease-specific patient-reported instrument and was therefore selected as the main measure of the TRANSTAR impact on patients’ quality of life.

##### EuroQoL-5-dimensions (EQ-5D and EQ-VAS) score

The EQ-5D is a validated generic health-related quality of life assessment yielding a patient health profile along five dimensions: mobility, self-care, usual activities, pain/discomfort, and anxiety/depression [19]. Each dimension is represented by one item with three response options: no problem, some problems, and severe problems. Responses to these five items can be normatively weighted to derive an EQ-5D utility score with a range of −0.594 to 1 (one being ultimate health). The EQ-5D score was assessed according to its original derivations, with an improvement in QoL expressed by an increase in numerical value of the score [23, 24]. A difference of ≥0.07 in EQ-5D utility has been identified as clinically important [25]. The EQ-VAS is a visual analog scale representing a single-item global quality of life assessment in which patients are asked to rate their current health on a scale from 0 (worst imaginable) to 100 (best imaginable) [26]. The EQ-5D and EQ-VAS were selected in this registry because of their usefulness in estimating the relative cost-effectiveness of a surgical intervention.

### Statistical methods

The completed questionnaires of the scoring systems were analyzed comparing 12-month follow-up with baseline data. Mean and median values were computed for all functional and quality of life scoring systems and were presented along with their 95 % CI. The collected patient data were analyzed using SPSS 19 (Statistical Package for the Social Sciences; SPSS, Chicago, IL, USA) software. For the comparisons between baseline and 12 months functional and quality of life score outcomes, the nonparametric Wilcoxon signed ranks test or *t* test were used where appropriate. The chi-square test or two-tailed Fisher exact test were used to compare qualitative data. All *p* values <0.05 were considered statistically significant.

## Results

### Demographics and baseline findings

Between January 2009 and January 2011, a total of 100 consecutive patients with ODS who underwent TRANSTAR in the 22 local investigating centers were enrolled in this cohort study. The median number of patients enrolled per an investigating center was 5 (range, 1–11). During the recruitment period, 11 centers recruited up to four patients, ten centers recruited 5–10 patients, and one center recruited 11 patients.

The majority of the patients were female (98 %). The mean age of the cohort was 60 (range, 27–82) years, and the mean body mass index (BMI) 27 (range, 18–45) kg/m^2^. Of these patients, 41 % were healthy, 45 % had mild systemic disease, 14 % had severe systemic disease, and no patients had severe systematic disease that was a constant threat to life. Previous abdominal surgery included hysterectomy in 32 %, hemorrhoidectomy in 13 %, and pelvic floor repair in 12 % of the cohort.

Prior to surgery for ODS, all 100 (100 %) patients underwent clinical anorectal examination and proctoscopy in the investigating centers. Consequently, a rectocele was documented in 93, an internal rectal prolapse in 77, and a muco-haemorrhoidal prolapse in 35 patients. Out of the 77 patients with an internal rectal prolapse, in 43 (56 %) patients was documented an internal recto-rectal prolapse, and in 34 (44 %) patients an internal recto-anal prolapse.

Additionally to the clinical anorectal examinations and proctoscopy, 97 patients had undergone diagnostic defecating imaging in the local investigating centers, either by defecography (68 %) or by MRI (29 %), which demonstrated the presence of a rectocele in 80 % and an internal rectal prolapse in 73 % of patients. Perineal descent was present in 19 % and a non-fixed enterocele in 14 % of patients. Only in three patients, the additional defecating imaging (defecography or MRI) was not performed based on the decision of the local investigators in line with local policy.

### Surgical findings

In the cohort, the mean hospitalization time was 4.36 ± 2.75 (range, 1–12) days and the mean operative time was 43.8 ± 13.9 (range, 25–90) min. The mean weight of recorded specimens was 37.8 ± 25.3 g (range, 10–98 g). Concomitant surgeries were infrequently reported and comprised removal of skin tag(s) in 5 %, diagnostic laparoscopy in 5 %, and excision of anal polyp(s) in 1 % of patients.

### Safety of the procedure

The safety analysis evaluated intra- and postoperative complications of all patients entered in the study, regardless of the completeness of other data or the length of follow-up. In total, 3 % intraoperative and 8 % postoperative complications contributing to the overall TRANSTAR-related morbidity rate of 11 % were recorded. Intraoperatively, the three (3 %) staple line complications included a partial dehiscence of the staple line in one (1 %) and spiraling of the staple line in two patients (2 %). The partial dehiscence of the staple line in one patient required immediate additional suturing with no further surgical re-intervention. The spiraling of the staple line in two patients required conservative treatment by observation and oral antibiotic medication with no further surgical treatment. Postoperatively, eight complications (8 %) occurred, including bleeding (5 %), urinary retention (2 %), and persistent pain (1 %). Prolonged persistent pain in one patient required removal of retained staples (agraphectomy). One patient with postoperative bleeding received conservative treatment, and in one patient the observed perirectal hematoma required surgical re-intervention. In three patients, self-limiting rectal bleeding was notified. Acute urinary retention occurred in two patients (2 %) requiring interventions without anesthesia. No major complications were documented and no mortality occurred. A complete breakdown of the complications is given in Table 1.

**Table 1:** Combined reporting of complications (operative, peri- and postoperative) in 100 patients entered into the European Transtar registry

TRANSTAR complications	Number of patients with complications	(%) among all patients
Bleeding	5	5
Staple line complications	3	3
Urinary retention	2	2
Persistent pain	1	1
Total	11	11

### Effectiveness of the procedure

#### Study scoring systems

Preoperatively and postoperatively fully completed questionnaires were analyzed at 12 months follow-up, and the completeness of data collection varied according to the scoring system used: KESS score 63 %, ODS score 65.0 %, St. Mark’s incontinence score 65 %, PAC-QoL score 65 %, EQ-5D score 64.0 %, and EQ-VAS scale 64.0 %. Consequently, the overall scoring response rate was in the very narrow range of 63–65 %.

**Fig. 1:**
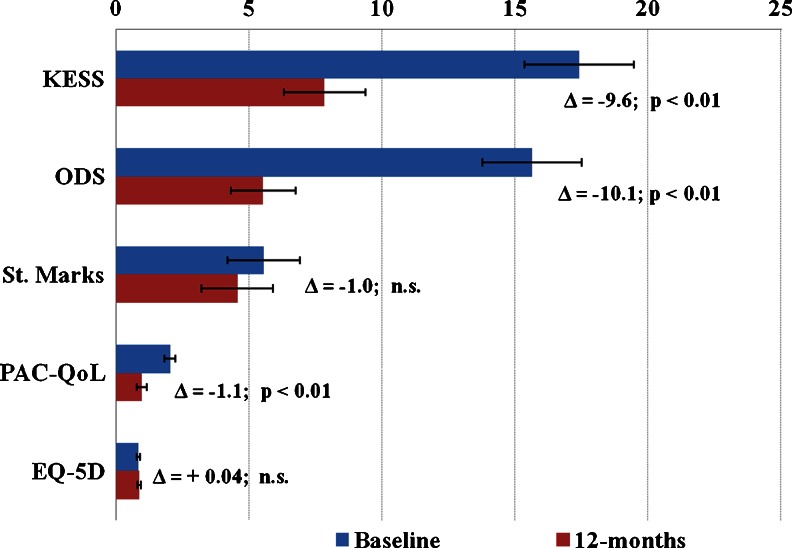
Comparisons of study scores mean values at baseline and 12 months postoperatively. Statistically significant improvements were observed in the KESS, ODS, and PAC-QoL scores at 12 months as compared with baseline (*Wilcoxon signed ranks test and paired samples *t* test, both *P* < 0.01)

**Fig. 2:**
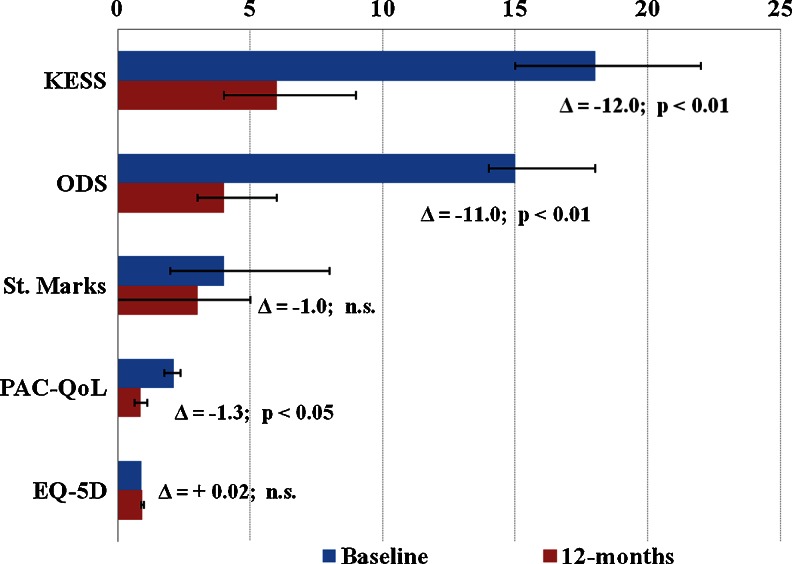
Comparisons of study scores median values at baseline and 12 months postoperatively. Statistically significant improvements were observed in the KESS, ODS, and PAC-QoL scores at 12 months as compared with baseline (*Wilcoxon signed ranks test and paired samples *t* test, both *P* < 0.01)

Changes in the assessed study scoring systems’ mean and median values between baseline and 12 months follow-up are presented in Figs. 1 and 2.

#### Functional scores

**Fig. 3:**
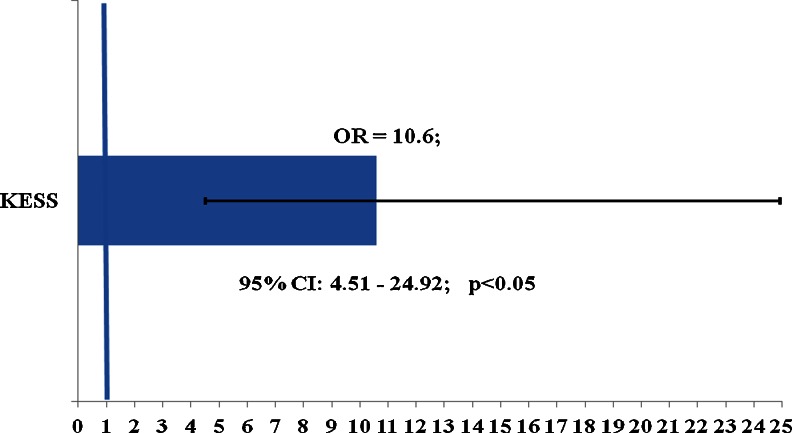
Odds ratio (OR) for ODS calculated from KESS score assuming the preoperative symptoms would remain unchanged in an untreated control group. TRANSTAR significantly reduce the risk of ODS by ten times (OR = 10.6) at 12 months postoperatively, when a median of ≥10 for KESS score was used as a cut-off criterion between constipated and non-constipated groups

As the primary functional endpoint in this study, the KESS constipation score was statistically significantly improved at 12 months postoperatively. The median score value improved from *18* [95 % CI: 15–22] to *6* [95 % CI: 4–9]; (*p* < 0.01), and the mean score value improved from *17.14* (SD 7.81); [95 % CI: 15.59–18.69] to *7.82* (SD 6.08); [95 % CI: 6.29–9.35]; (*p* < 0.01). Significant improvements were recorded in all individual score items’ mean values at 12 months compared to baseline (Table 2). In accordance with the KESS score validation report, a cut-off criterion of a median KESS score ≥10 was used to differentiate constipated patients from healthy individuals [20]. A total of 80 % of patients presented a median KESS score change from ≥10 at baseline to <10 at 12 months follow-up. The calculated odds ratio showed that TRANSTAR statistically significantly reduced the risk of obstructive defecation at 12 months follow-up, by more than ten times (OR = *10.6*; [95 % CI: 4.51–24.92]), and presented in the Fig. 3. The number needed to treat (NNT) for this benefit at 12 months follow-up was 2.

**Table 2:** The individual KESS score items mean values at baseline and 12 months postoperatively. Statistically significant improvements were observed in all score items

KESS score items	Baseline mean (SD)	12-months mean (SD)	Difference of mean	*P* value
Duration of constipation	1.95 (1.54)	1.40 (1.60)	−0.55	*p* < 0.01*
Laxative use	1.19 (1.12)	0.68 (0.86)	−0.51	*p* < 0.01*
Frequency of bowel movement	0.52 (0.72)	0.19 (0.47)	−0.33	*p* < 0.01*
Unsuccessful evacuatory events	1.29 (0.94)	0.40 (0.71)	−0.89	*p* < 0.01*
Feeling of incomplete evacuation	2.68 (1.23)	1.10 (1.20)	−1.59	*p* < 0.01*
Abdominal pain	1.54 (1.27)	0.76 (0.90)	−0.78	*p* < 0.01*
Bloating	1.10 (0.95)	0.73 (0.70)	−0.37	*p* < 0.01*
Enemas/digitation	1.87 (1.63)	0.40 (0.93)	−1.48	*p* < 0.01*
Time taken in lavatory/attempt	1.32 (0.78)	0.62 (0.69)	−0.70	*p* < 0.01*
Difficulty evacuating	2.86 (1.26)	1.00 (1.15)	−1.86	*p* < 0.01*
Stool consistency (without laxatives)	1.10 (0.93)	0.56 (0.67)	−0.54	*p* < 0.01*
Total score: mean (SD)	17.14 (7.81)	7.82 (6.08)	−9.57	*p* < 0.01*

*Wilcoxon signed ranks test and paired samples *t* test, both *P* < 0.01

As the secondary functional endpoint in this study, the ODS constipation score was statistically significantly improved at 12 months postoperatively. The median score value from *15* [95 % CI: 14–18] to *4* [95 % CI: 3–6] (*p* < 0.01), and the mean score value improved from *15.65* (SD 7.63); [95 % CI: 14.13–17.16] to *5.52* (SD 4.86); [95 % CI: 4.31–6.72]; (*p* < 0.01). Significant improvements were recorded in all individual score items’ mean values between baseline and 12 months postoperatively (Table 3).

**Table 3:** The individual ODS score items mean values at baseline and 12 months postoperatively. Statistically significant improvements were observed in all score items

ODS score items	Baseline mean (SD)	12-months mean (SD)	Difference mean	*P* value
Defecation frequency	1.08 (1.11)	0.34 (0.62)	−0.74	*p* < 0.01*
Intensive straining	1.51 (0.71)	0.55 (0.64)	−0.95	*p* < 0.01*
Time spent on defecation	1.52 (0.69)	0.75 (0.64)	−0.77	*p* < 0.01*
Feeling of incomplete defecation	2.20 (1.00)	0.91 (1.06)	−1.29	*p* < 0.01*
Pain	1.91 (1.18)	0.46 (0.79)	−1.45	*p* < 0.01*
Impact on daily activity	1.91 (2.16)	0.89 (1.42)	−1.02	*p* < 0.01*
Use of laxatives	2.05 (2.56)	1.09 (2.04)	−0.95	*p* < 0.01*
Use of enemas	1.26 (1.94)	0.28 (1.08)	−0.98	*p* < 0.01*
Digital assistance	2.20 (2.61)	0.25 (0.88)	−1.95	*p* < 0.01*
Total score: mean (SD)	15.65 (7.52)	5.52 (4.86)	−10.12	*p* < 0.01*

*Wilcoxon signed ranks test and paired samples *t* test, both *P* < 0.01

St. Mark’s incontinence score improved numerically, but nonsignificantly, with a median score value of *4* [95 % CI: 3–7] at baseline, and of *3* [95 % CI: 1–5] at 12 months follow-up; (*p* = n.s). Nonsignificant improvement was seen also in the mean score value from *5.93* (SD 5.63); [95 % CI: 4.81–7.04]; to *4.55* (SD 5.42); [95 % CI: 3.21–5.89]; (*p* = 0.184) at 12 months postoperatively as compared with baseline.

#### Quality of life

The disease-specific PAC-QoL score was statistically significantly improved at 12 months postoperatively. The median disease-specific PAC-QoL score improved statistically significantly from *2.10* [95 % CI: 1.85–2.30] to *0.86* [95 % CI: 0.65–1.11]; (*p* < 0.01), and the mean score value from *2.0* (SD 0.82); [95%CI: 1.83–2.16;] to *0.95* (SD 0.71); [95 % CI: 0.77–1.33]; (*p* < 0.01). Significant improvements were recorded in all four individual score domains (physical discomfort, psychosocial discomfort, worries and concerns, satisfaction) between baseline and 12 months follow-up (Table 4).

**Table 4:** The individual PAC QoL score domains mean values at baseline and 12 months postoperatively. Statistically significant improvements were observed in all score domains

PAC-QoL score domains	Baseline mean (SD)	12-months mean (SD)	Difference mean	*P* value
Physical discomfort	1.92 (1.11)	0.84 (0.84)	1.04	*p* < 0.01*
Psychosocial discomfort	1.37 (0.96)	0.63 (0.75)	0.75	*p* < 0.01*
Worries and concerns	1.93 (0.94)	0.94 (0.78)	1.06	*p* < 0.01*
Satisfaction	2.78 (0.95)	1.39 (0.95)	1.45	*p* < 0.01*
Total score: mean (SD)	2.00 (0.82)	0.95 (0.71)	−1.09	*p* < 0.01*

*Wilcoxon signed ranks test and paired samples *t* test, both *P* < 0.01

The generic quality of life score EQ-5D improved numerically, but nonsignificantly, with a median score value of *0.89* [95 % CI: 0.88–0.90] compared to *0.91* [95 % CI: 0.89–0.1.0] at 12 months follow-up (*p* = n.s). A nonsignificant improvement was seen also in the mean score values from *0.82* (SD 0.22); [95 % CI: 0.77–0.86]; to *0.86* (SD 0.24); [95 % CI: 0.79–0.92]; (*p* = 0.184) at 12 months postoperatively as compared with baseline.

The mean EQ-VAS score improved nonsignificantly from *54.26* (SD 26.45); [95 % CI: 49.07–59.61] to *56.30* (29.93); [95 % CI: 48.82–63.77] (*p* = n.s), and the median score value did not change between baseline and 12 months follow-up.

#### Fecal incontinence, urgency, and pain

The postoperative risks of fecal incontinence, defecatory urgency, and abdominal pain were evaluated according to the specific questions derived from the validated KESS and St. Mark’s scores assuming the preoperative symptoms would remain unchanged in an untreated control group. The postoperatively documented adverse events were also taken into account.

Significant reductions in fecal incontinence (OR = 0.30; [95 % CI: 0.14–0.65]) and abdominal pain (OR = 0.32; [95 % CI: 0.15–0.67]) were observed at 12 months follow-up. In contrast, a nonsignificant increase in the chance of experiencing defecatory urgency (OR = 1.15; [95 % CI: 0.55–2.40]) was noted at 12 months follow-up. Preoperatively, 31 % of the enrolled patients experienced urgency symptoms. At 12 months follow-up, the urgency symptoms were documented in 34 % of the surgically treated patients.

**Fig. 4:**
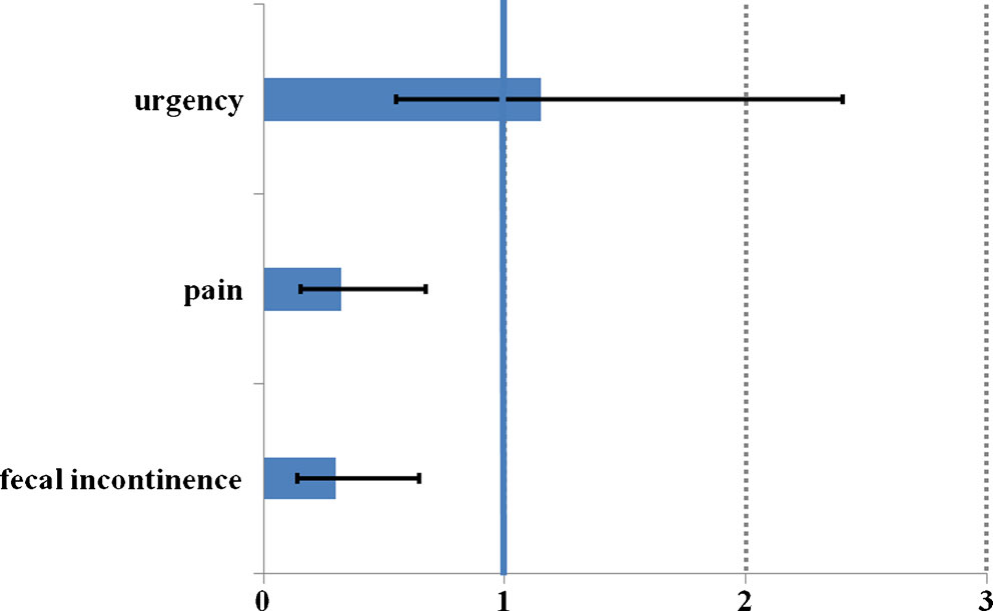
Odds ratio (OR) for fecal incontinence, pain, and urgency calculated from KESS score and St. Marks score assuming the preoperative symptoms would remain unchanged in an untreated control group. TRANSTAR significantly reduce the risk of fecal incontinence (OR = 0.30) and abdominal pain (OR = 0.32) and nonsignificantly increase the risk of urgency (OR = 1.15) at 12 months postoperatively

The odds ratios for fecal incontinence, urgency, and abdominal pain at 1-year follow-up compared to baseline are presented in Fig. 4.

## Discussion

In this multicenter study, 22 individual surgeons assessed the short-term safety, clinical effectiveness, and quality of life outcomes after TRANSTAR performed in the real-world clinical setting throughout Europe. The primary functional endpoint was the validated KESS constipation score, which improved statistically significantly on all individual score items between baseline and 12 months follow-up. Consequently, for the vast majority of patients defecation difficulties improved postoperatively, with 80 % changing from constipated patients to healthy individuals, according to the cut-off criterion from the score validation report [20]. The risk assessment based on the changes in the validated KESS score showed a highly significant, more than ten times (OR = 10.6) reduction in the chance to experience symptoms of ODS at 12 months postoperatively, and the number of patients needed to treat for reaching this benefit was very low (NNT = 2). This reflects the real-world effectiveness of this transanal stapling procedure in the treatment of ODS. To our knowledge, this is the first study where the validated KESS score has been used as a primary functional endpoint to assess the clinical effectiveness of TRANSTAR.

Although not validated, the in the literature broadly reported Longo’s ODS score was the secondary functional endpoint in this cohort. It improved significantly, reflected by the decrease in its overall mean and median values (−10.12 and −11, respectively) at 12 months follow-up as compared to baseline. In the literature, Wolff et al. [27] reported a significant improvement in the ODS score after TRANSTAR, expressed as a decrease (−11) in its median score value, at 6 months postoperatively. Savastano et al. [28] recently reported a significant improvement in ODS score at 6 months follow-up, expressed as a decrease (−10.8) in its mean score value. Lenisa et al. [16] reported an even larger improvement in ODS score at 1 year after TRANSTAR, expressed as a decrease in mean score (−14.6). In the current study, the observed mean and median ODS score improvements at 12 months follow-up appeared to be very similar to the significant ODS changes reported in recently published trials. This reinforces the clinical effectiveness of TRANSTAR as assessed by the KESS score in this study.

The impressive improvements observed in the functional constipation scores are supported by the significant improvement in constipation-specific quality of life as measured by the validated PAC-QoL score. The disease-specific PAC-QoL score improved statistically significantly on all four quality of life domains: patient’s satisfaction, physical discomfort, psychosocial discomfort, and worries and concerns, reaching statistical significance at 12 months follow-up as compared to baseline. The generic quality of life score EQ-5D improved numerically without reaching statistical significance, probably due to its relatively high baseline value (0.82) in this cohort.

In the literature, Jane et al. reported significant improvements in the validated quality of life instruments EQ-5D and PAC-QoL at 12 months follow-up after STARR for ODS [15]. It has also been reported that patient quality of life significantly improved after TRANSTAR, particularly on the mental components of the FIQL and SF36 instruments [27]. In a recent randomized controlled trial, Bocassanta et al. [29] reported that the SF36 Health Survey score significantly improved after STARR and TRANSTAR, and that TRANSTAR patients showed a trend towards better satisfaction. The patient reported outcomes in this study reinforce evidence from previous series that transanal stapling procedures for ODS significantly improve constipation-related patient quality of life.

Fecal incontinence has preoperatively been seen as a typical symptom of ODS, especially in patients with grade III rectal intussusception and rectoceles [30], having negative impact on patients’ quality of life. Moreover, fecal incontinence also negatively affects well-being in patients with other anorectal disorders [31–34]. However, in recently published studies, it was observed that fecal incontinence usually improved after transanal stapling procedures for ODS. In the STARR Registry, Jane et al. [16] reported a significant improvement in the validated Cleveland Clinic Fecal Incontinence score at 12-month follow-up as compared with baseline, and this was mirrored by a significant improvement in patient-reported symptoms of incontinence/soiling, captured as a component of the symptom severity score [15]. Wolf et al. [27] reported that all preoperatively incontinent patients included in their trial were continent at 6 months after TRANSTAR. Lenisa et al. [16] reported a 41 % incontinence cure rate after TRANSTAR at 1-year follow-up as compared to baseline. Isbert et al. [35] compared STARR and TRANSTAR at 1-year follow-up, reporting that neither technique compromised anal sphincter function, and that the postoperatively gained ability to effectively evacuate the rectum may rather help in reducing incontinence episodes. Additionally, it has been observed that patients in whom fecal incontinence occurred as a new symptom after TRANSTAR tended to be those with a preexisting asymptomatic incontinence, appearing after anatomic restoration [27, 35].

In this cohort study, the risk assessment (OR = 0.30) showed a significant reduction in the chance to experience fecal incontinence at 12 months after TRANSTAR as compared to baseline. The real-world data from this study reinforces the published evidence, showing that fecal incontinence combined with an intact anal sphincter, observed preoperatively, may not be a contraindication for proposing transanal surgery for ODS in itself [15, 16, 27, 35].

As a component of impaired continence, defecatory urgency has been seen as a common postoperative finding after surgery using transanal stapling. Urge symptoms were reported after a low anterior resection for rectal cancer and observed up to 12 months postoperatively, without requiring any additional therapy [36]. In the treatment of ODS, defecatory urgency was often recorded after STARR and after TRANSTAR [15, 16, 37]. In the majority of patients, the urge symptoms disappeared postoperatively without the need for any additional therapy, irrespective of whether it was observed as a new onset of the symptom or it already persisted as a symptom of the disease at baseline [16, 27, 35]. However, in the European STARR registry, 26.8 % of patients still complained of urgency at 1-year follow-up [15]. Renzi et al. [17] and Savastano et al. [28] reported an incidence of urge symptoms of 17.2 and 18.7 %, respectively, at 6 months after TRANSTAR. The reduction in the rectal ampula volume and modification of rectal sensitivity, as the natural consequences of the TRANSTAR technique, appear to be the reason for the increased risk of urge symptoms postoperatively [16, 27, 35].

In this study, the risk assessment showed a nonsignificant increase (OR = 1.15) in the chance to experience defecatory urgency at 12 months follow-up as compared to baseline. Consequently, St. Mark’s incontinence score, which includes a specific urgency measure, was the only functional score in this study that improved nonsignificantly at 12 months follow-up, probably due to a negative impact of urgency on the overall score. However, patients should be informed about the risk of urge symptoms, possibly for a prolonged period of time after transanal stapling procedures for ODS.

In recent series, Wolf et al. [27] and Martelucci et al. [37] reported TRANSTAR-related morbidity rates of 8 and 16 %, respectively. Renzi et al. [17] reported up to 31 % early complications and 24 % late complications after TRANSTAR.

The safety analysis in this study showed a lower percentage of intra- and postoperative complications recorded, contributing to the TRANSTAR-related morbidity rate of 11 %. Intraoperatively, a lower proportion of 3 % complications such as a partial dehiscence and spiraling of the staple line were recorded in this study, without any need for postoperative therapy. Lenisa et al. reported moderate intraoperative difficulties with TRANSTAR, including 5 % partial dehiscence and 4 % spiraling of the staple line, concluding that the spiral resection of the rectum during TRANSTAR may result from technical mistakes, such as inappropriate traction on the parachute stitches at the top of the internal rectal prolapse, or from a larger amount of the rectal wall incorporated into the jaw of the device [16]. Accordingly, the immediate observation of the staple line to detect possible staple line leakages has been recommended [16].

Postoperatively, 5 % postoperative bleeding, 2 % urinary retention, and 1 % persistent pain were recorded in this study. The majority of patients with postoperative bleeding experienced self-limiting bleeding episodes, and one patient needed an additional surgical intervention for the revision of a perirectal hematoma.

Postoperative bleeding has been presented in various series in the literature as occurring in 1.5 to 7 % of patients after TRANSTAR. Episodes are usually self-limiting, but if needed they can be managed successfully with conservative treatment or in rare cases by revision of the staple line [16, 35, 38].

According to recent series, the incidence of acute urinary retention varies from 1.2 to 10.3 % and usually requires short-term urinary catheterization [27, 37, 38], which is in accordance with the two patients with urinary retention after TRANSTAR in this study.

Moreover, it has been reported that some patients might suffer from persistent anorectal pain after transanal stapling procedures, caused by a proctitis due to retained staples [39]. The removal of the retained staples led to resolution of the symptoms in previously published series [16, 27]. Similarly, prolonged anorectal pain was observed postoperatively in one patient in this study, and the surgical removal of the retained staples contributed to its resolution.

However, abdominal pain can also be observed preoperatively, as part of the ODS symptoms [2]. In this study, the risk assessment showed a significant reduction in the chance of experiencing abdominal pain (OR = 0.32) at 12 months follow-up as compared to baseline. The data from this study support previously published series concluding that retained staples might occasionally be a cause of persistent pain after TRANSTAR, but overall, the risk of postoperative abdominal pain as a symptom of ODS seemed to be significantly reduced.

No major or life-threatening complications after TRANSTAR were recorded in this study, although they have been reported in the literature. A rectal perforation was reported by Schulte et al. [40], in which intraperitoneal occurring emphysema was treated conservatively. A large hematoma in the mesorectum extending towards both kidneys requiring a laparotomy was reported by Gelos et al. after TRANSTAR [41]. Martelucci et al. [37] reported a rectal perforation and a rectovaginal fistula after TRANSTAR, complications which were also reported for the original STARR technique [37, 42]. Savastano et al. [28] reported a hemoperitoneum after TRANSTAR, which was treated with synchronous colostomy and subsequent recanalization.

Finally, if compared with the safety data from previously published trials, the TRANSTAR-related morbidity profile observed in this study appears to be acceptable.

Data incompleteness could be seen as a limitation of this study because the questionnaires used for the 12-months analysis had a completeness range of 63–65 %, according to the scoring system used. The reason for this is thought to be that the majority of the study population consisted of elderly women, and some felt embarrassed answering detailed questions concerning their sexual functioning and defecation disorders. Accordingly, some patients provided answers only for specific chapters of the questionnaires, or they refused to fill out the questionnaires preoperatively, and only did so after surgery. However, this is not unexpected for an observational, real-world study. For example, in the STARR registry, it was reported that for the patients eligible for 12-month follow-up analysis, the completeness of data collection varied from 41 to 64 % according to the scoring system used [16]. Isbert et al. [35] reported in their comparative observational trial that 58 % of patients in the STARR group and 46 % of patients in the TRANSTAR group attended their follow-up assessments at 12 months.

Furthermore, it can be argued that the relatively short follow-up of only 12 months postoperatively presents a limitation of this study and that it needs to be proven that the current functional results are sustainable in the longer term. Indeed, outcomes from randomized clinical trials from 2 and 3 years after TRANSTAR have recently been published. Renzi et al. [43] reported a longer maintenance of symptom relief in patients who had undergone TRANSTAR compared to those who underwent STARR at 2-year follow-up. Bocassanta et al. [29] were able to demonstrate a significantly lower incidence of fecal urgency and internal prolapse recurrence as two major benefits of TRANSTAR when compared to STARR at 3-year follow-up. The authors concluded that the prolapse recurrence rate was significantly lower in favor of TRANSTAR compared to STARR, probably due to the larger extent of rectal wall resection [29]. It can be concluded that the impressive functional results achieved in the relatively artificial environment of these randomized clinical trials need to be confirmed in broader surgical practice with larger patient populations.

## Conclusion

TRANSTAR facilitated a tailored, real circumferential full-thickness rectal resection, performed in the real-world clinical setting, without any major complications recorded. It led to statistically significantly improved patient functional and quality of life outcomes at 12 months postoperatively, as measured by validated constipation-specific scoring systems such as the KESS and PAC-QoL scores.
